# Development and validation of a model for predicting the expression of Ki-67 in pancreatic ductal adenocarcinoma with radiological features and dual-energy computed tomography quantitative parameters

**DOI:** 10.1186/s13244-024-01617-8

**Published:** 2024-02-14

**Authors:** Youjia Wen, Zuhua Song, Qian Li, Dan Zhang, Xiaojiao Li, Jiayi Yu, Zongwen Li, Xiaofang Ren, Jiayan Zhang, Qian Liu, Jie Huang, Dan Zeng, Zhuoyue Tang

**Affiliations:** Chongqing General Hospital, No.118, Xingguang Avenue, Liangjiang New Area, Chongqing, China

**Keywords:** Dual-energy computed tomography (DECT), Pancreatic ductal adenocarcinoma (PDAC), Ki-67, Prognosis, Nomogram

## Abstract

**Objective:**

To construct and validate a model based on the dual-energy computed tomography (DECT) quantitative parameters and radiological features to predict Ki-67 expression levels in pancreatic ductal adenocarcinoma (PDAC).

**Materials and methods:**

Data from 143 PDAC patients were analysed. The variables of clinic, radiology and DECT were evaluated. In the arterial phase and portal venous phase (PVP), the normalized iodine concentration (NIC), normalized effective atomic number and slope of the spectral attenuation curves were measured. The extracellular volume fraction (ECVf) was measured in the equilibrium phase. Univariate analysis was used to screen independent risk factors to predict Ki-67 expression. The Radiology, DECT and DECT–Radiology models were constructed, and their diagnostic effectiveness and clinical applicability were obtained through area under the curve (AUC) and decision curve analysis, respectively. The nomogram was established based on the optimal model, and its goodness-of-fit was assessed by a calibration curve.

**Results:**

Computed tomography reported regional lymph node status, NIC of PVP, and ECVf were independent predictors for Ki-67 expression prediction. The AUCs of the Radiology, DECT, and DECT–Radiology models were 0.705, 0.884, and 0.905, respectively, in the training cohort, and 0.669, 0.835, and 0.865, respectively, in the validation cohort. The DECT–Radiology nomogram was established based on the DECT–Radiology model, which showed the highest net benefit and satisfactory consistency.

**Conclusions:**

The DECT–Radiology model shows favourable predictive efficacy for Ki-67 expression, which may be of value for clinical decision-making in PDAC patients.

**Critical relevance statement:**

The DECT–Radiology model could contribute to the preoperative and non-invasive assessment of Ki-67 expression of PDAC, which may help clinicians to screen out PDAC patients with high Ki-67 expression.

**Key points:**

• Dual-energy computed tomography (DECT) can predict Ki-67 in pancreatic ductal adenocarcinoma (PDAC).

• The DECT–Radiology model facilitates preoperative and non-invasive assessment of PDAC Ki-67 expression.

• The nomogram may help screen out PDAC patients with high Ki-67 expression.

**Graphical Abstract:**

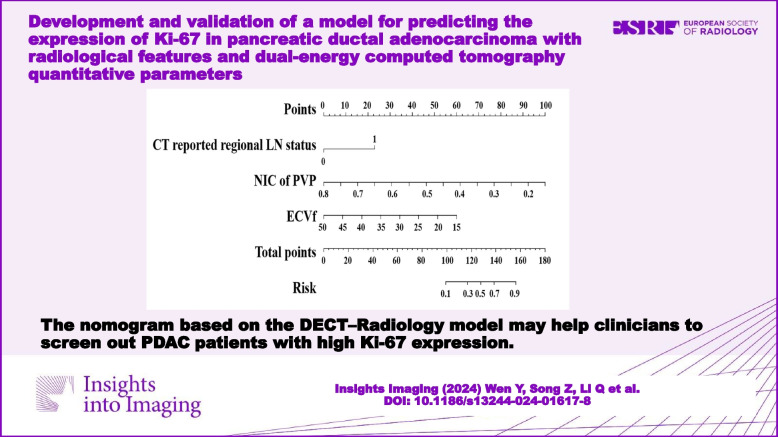

**Supplementary Information:**

The online version contains supplementary material available at 10.1186/s13244-024-01617-8.

## Introduction

Pancreatic ductal adenocarcinoma (PDAC), an extremely malignant and invasive tumour, is estimated to be the second principal cause of cancer-related death globally in 2030 [1]. PDAC progresses rapidly, and most patients already have lymph nodes or distant metastases when the time of diagnosis is confirmed [2, 3]. Although advances in multimodal therapy have improved, the 5-year survival rate for PDAC has not increased obviously and remains at approximately 11% [4–6]. Ki-67 possesses high expression in the majority of proliferating cells except for G0, which is one of the important immunohistochemical markers designating tumour heterogeneity and cell growth and can accurately reflect the proliferative activity of tumour cells [7]. Previous inquiries have suggested a negative relationship between the expression level of Ki-67 and the differentiation degree of PDAC tumours, and PDAC patients with high Ki-67 expression levels were more likely to develop metastases in regional lymph nodes (LNs) and the liver [8–10]. Ki-67 was also an independent risk factor for the poor prognosis of PDAC and the recurrence within 1 year after pancreaticoduodenectomy [8, 10]. Therefore, accurately evaluating the degree of Ki-67 expression is important for judging the prognosis of PDAC.

Currently, the expression of Ki-67 can only be determined by immunohistochemistry (IHC) on puncture or surgical specimens. Nevertheless, a single puncture may not always be successful in sampling, and the resulting multiple punctures may cause multiple injuries and inconveniences to the patient [11]. Furthermore, biopsy and surgical specimens are invasive methods that may induce tumour ulcers or breaches to increase the risk of tumour spread. Hence, it is crucial to create a noninvasive, convenient and effective measure to predict the level of Ki-67 expression in PDAC.

Dual-energy computed tomography (DECT) uses two X-ray sources with different energy levels to image objects and has shown prospects for clinical applications in the detection and characterization of tumours. Various quantitative metrics derived from DECT, such as normalized iodine concentration (NIC), normalized effective atomic number (NZeff) and slope of the spectral HU curve (λHU), have been gradually applied to predict the Ki-67 in various types of solid tumours, including laryngeal squamous cell carcinoma, breast cancer, and gastric adenocarcinoma [12–14]. As an emerging quantitative DECT parameter, extracellular volume fraction (ECVf) has also been applied to evaluate the efficacy of radiotherapy and survival analysis with neoadjuvant chemotherapy in PDAC [15, 16]. However, the association between DECT-derived parameters and the degree of Ki-67 expression in PDAC patients is ill-defined and infrequently reported. Meanwhile, preoperative radiological features such as the presence of focal pancreatic parenchymal atrophy, pancreatic duct expansion, peritumoural vessel invasion and extrapancreatic perineural invasion were found to indicate the poor prognosis of PDAC patients [17–20], and have an important impact on the treatment decision of PDAC.

In this research, we supposed that DECT quantitative parameters could predict Ki-67 expression in PDAC. To prove our assumption, we attempt to establish a model binding DECT quantitative parameters and radiological features to recognize patients with high Ki-67 expression and visualize it as a nomogram for assisting in the realization of individualized clinical decision-making in PDAC patients.

## Materials and methods

### Patients

**Fig. 1:**
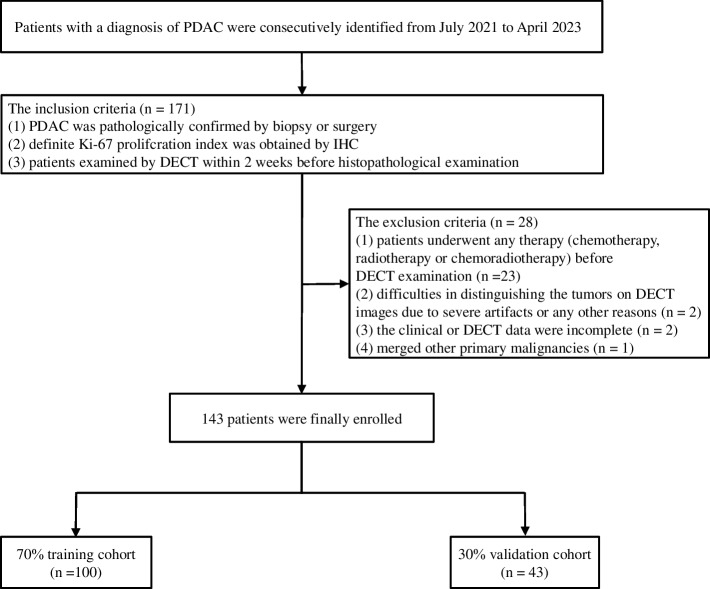
Flowchart of the participant inclusion process. PDAC, pancreatic ductal adenocarcinoma; IHC, immunohistochemistry; DECT, dual-energy computed tomography

The research obtained consent from the Ethics Committee of the Chongqing General Hospital. Owing to the retrospective nature of the study, we waived the requirement for informed consent from the participants. Data on patients with pathologically confirmed PDAC and examined Ki-67 were continuously collected at Chongqing General Hospital from July 2021 to April 2023 based on inclusion and exclusion criteria (Fig. 1). The inclusion criteria were as follows: (1) PDAC was confirmed by biopsy or surgery; (2) Ki-67 was examined by IHC; and (3) DECT was performed within 14 days of the examination of Ki-67. The exclusion criteria were as follows: (1) neoadjuvant chemotherapy or radiotherapy before Ki-67 examination; (2) unsatisfactory image quality for analysis; (3) merged other primary malignancies; and (4) partially missing clinical, pathological, surgical or imaging data.

Eventually, 143 patients (81 men and 62 women; mean age ± SD, mean age 62.57 ± 0.86 years) were recruited and were further randomly divided into the training and validation cohorts at a ratio of 7:3 (*n* = 100, 70.0%; 43, 30.0%).

### Ki-67 index measurement

The Ki-67 of the 143 patients was examined by IHC performed on surgical specimens (52.4%) and percutaneous or transgastric core biopsy specimens (47.6%). All specimens were from primary pancreatic tumours. Cells with brown nuclei were defined as positive. The Ki-67 was 0–100% based on the proportion of 1000 positive cells randomly selected at 200 × magnification. Since the optimal cut-off value for the Ki-67 low or high differentiation set of PDAC has not been determined, 50% was chosen as the cut-off on the basis of previous studies [8]. The samples were divided into a low Ki-67 expression set (Ki-67 ≤ 50%) and a high Ki-67 expression set (Ki-67 > 50%).

### DECT image acquisition

Contrast-enhanced abdominal scans were performed using a DECT (IQon spectral CT, Philips Healthcare). All DECT scans were performed by scanners using a standard protocol, with details described in the Supplementary material.

### Radiology and DECT candidate variables

The radiological candidate variables of PDAC included tumour location, CT-reported T stage, CT-reported regional LN status, atrophy of the pancreatic parenchyma, expansion of the main pancreatic duct, blood vessels invasion and extrapancreatic perineural invasion. The DECT candidate variables of PDAC included NIC, NZeff and λHU in the arterial phase (AP) and portal vein phase (PVP), and the extracellular volume fraction (ECVf) in the equilibrium phase (EP). Details are described in the Supplementary material.

### Construction of the models and nomogram

The differences in variables between low and high Ki-67 expression sets were contrasted with univariate analysis first. The independent risk factors for predicting the Ki-67 expression of PDAC patients were ascertained after forward stepwise binary logistic regression from the significant parameters (*p* < 0.05) in the training cohort. Next, the Radiology, DECT and DECT–Radiology models were built depending on the independent hazard elements. The above models were independently proven in the validation cohort. The diagnostic effectiveness and clinical applicability of the three models were obtained through receiver operating characteristic curve (ROC) and decision curve analysis (DCA). The contrast for the area under the curve (AUC) was determined through the Delong test. Finally, a nomogram was developed from the model with the highest diagnostic performance. The goodness-of-fit of the nomogram was evaluated through the calibration curve.

### Statistical analysis

All calculations and statistical analyses were executed in R software (https://www.r-project.org/), MedCalc (version 18.2.1, MedCalc Software) and SPSS software (version 26.0, SPSS, IBM). The Shapiro–Wilk test was utilized to verify the data normality. The data were demonstrated as the mean ± standard deviation (SD) for normally distributed data and as the median (25th, 75th percentiles) for non-normally distributed data. Continuous variables were estimated through a two-sample *t* test or Mann–Whitney *U* test. Categorical variables were estimated through the chi-square test. A two-sided *p* value < 0.05 declared statistical significance.

## Results

### Radiological features and DECT quantitative parameters

Table 1 displays variables from the clinic, radiology and DECT of the study. In the training cohort, the CT-reported regional LN status and all DECT quantitative parameters demonstrated significant differences with *p* < 0.05. Table 2 displays the representation of DECT quantitative parameters in the training cohort, including the mean values of λHU of PVP and the median values of NIC of AP, λHU of AP and NZeff of AP, NIC of PVP, NZeff of PVP and the ECVf. The above DECT quantitative parameters of the low Ki-67 expression set were significantly higher than those of the high Ki-67 expression set (*p* < 0.05). Statistically significant differences were not found in any clinical characteristics, tumour location, CT-reported T stage, parenchyma atrophy, pancreatic duct expansion or vessels invasion between the low Ki-67 expression set and the high Ki-67 expression set (*p* > 0.05).

**Table 1:** Variables of clinic, radiology and DECT in the training and validation cohorts

Variables	Training cohort (*n* = 100)	Validation cohort (*n* = 43)
**Clinical characteristics**		
Gender (%)		
Male	56 (56.0)	25 (58.1)
Female	44 (44.0)	18 (41.9)
Age (mean ± SD)	62.60 ± 10.55	62.49 ± 9.74
BMI (mean ± SD)	22.31 ± 2.76	21.57 ± 3.33
CEA (%)		
Normal	69 (69.0)	36 (83.7)
Elevated	31 (31.0)	7 (16.3)
CA-125 (%)		
Normal	61 (61.0)	30 (69.8)
Elevated	39 (39.0)	13 (30.2)
CA-199 (%)		
Normal	20 (20.0)	7 (16.3)
Elevated	80 (80.0)	36 (83.7)
**Radiological features**		
Tumour location (%)		
Head and neck	70 (70.0)	27 (62.8)
Body and tail	30 (30.0)	16 (37.2)
CT-reported T stage (%)		
T1–T2	54 (54.0)	23 (53.5)
T3–T4	46 (46.0)	20 (46.5)
CT-reported regional LN status (%)		
Negative	49 (49.0)	19 (44.2)
Positive	51 (51.0)	24 (55.8)
Parenchyma atrophy (%)		
Negative	63 (63.0)	20 (46.5)
Positive	37 (37.0)	23 (53.5)
Pancreatic duct expansion (%)		
Negative	32 (32.0)	17 (39.5)
Positive	68 (68.0)	26 (60.5)
Vessels invasion (%)		
Negative	72 (72.0)	32 (74.4)
Positive	28 (28.0)	11 (25.6)
Extrapancreatic perineural invasion status (%)		
Negative	33 (33.0)	12 (27.9)
Positive	67 (67.0)	31 (72.1)
**DECT quantitative parameters**		
NIC of AP	0.09 ± 0.04	0.08 ± 0.03
NZeff of AP	0.68 ± 0.04	0.68 ± 0.05
λHU of AP	1.37 ± 0.52	1.22 ± 0.50
NIC of PVP	0.38 ± 0.11	0.37 ± 0.07
NZeff of PVP	0.87 ± 0.05	0.86 ± 0.03
λHU of PVP	2.30 ± 0.70	2.09 ± 0.54
ECVf (%*)	36.08 ± 6.52	35.18 ± 6.69

The % value reflects the composition ratio

The %* value reflects the percentages

*AP* arterial phase, *DECT* dual-energy computed tomography, *ECVf* the extracellular volume fraction, *LN* lymph node, *NIC* normalized iodine concentration, *NZeff* normalized effective atomic number, *PVP* portal venous phase, *SD* standard deviation, *λHU* slope of the spectral Hounsfield unit curve

**Table 2:** Univariate analysis for the variables of clinic, radiology and DECT for differentiating between low and high Ki-67 expression sets in the training cohort

Variables	Low Ki-67 expression set (Ki-67 ≤ 50%, *n* = 77)	High Ki-67 expression set (Ki-67 > 50%, *n* = 23)	F/Z/χ^2^	*p *value
BMI	22.40 ± 2.84	22.00 ± 2.50	2.112	0.538
CEA (%)			0.337	0.562
Normal	52 (67.5)	17 (73.9)		
Elevated	25 (32.5)	6 (26.1)		
CA-125 (%)			0.978	0.323
Normal	49 (63.6)	12 (52.2)		
Elevated	28 (36.4)	11 (47.8)		
CA-199 (%)			3.391	0.066
Normal	19 (24.7)	1 (4.3)		
Elevated	58 (75.3)	22 (95.7)		
Tumour location (%)			0.325	0.568
Head and neck	55 (71.4)	15 (65.2)		
Body and tail	22 (28.6)	8 (34.8)		
CT-reported T stage (%)			0.076	0.782
T1–T2	41 (53.2)	13 (56.5)		
T3–T4	36 (46.8)	10 (43.5)		
CT-reported regional LN status (%)			10.36	< 0.001
Negative	45 (58.4)	4 (17.4)		
Positive	32 (41.6)	19 (82.6)		
Parenchyma atrophy (%)			1.502	0.220
Negative	51 (66.2)	12 (52.2)		
Positive	26 (33.8)	11 (47.8)		
Pancreatic duct expansion (%)			0.034	0.854
Negative	25 (32.5)	7 (30.4)		
Positive	52 (67.5)	16 (69.6)		
Vessels invasion (%)			0.088	0.766
Negative	56 (72.7)	16 (69.6)		
Positive	21 (27.3)	7 (30.4)		
Extrapancreatic perineural invasion status (%)			0.008	0.928
Negative	51 (66.2)	15 (65.2)		
Positive	26 (33.8)	8 (34.8)		
NIC of AP	0.09 (0.07, 0.12)	0.06 (0.04, 0.08)	− 3.051	0.002
NZeff of AP	0.66 (0.64, 0.67)	0.64 (0.61, 0.65)	− 1.994	0.046
λHU of AP	1.09 (0.78, 1.42)	0.77 (0.58, 1.03)	− 3.94	< 0.001
NIC of PVP	0.33 (0.30, 0.39)	0.24 (0.22, 0.29)	− 4.902	< 0.001
NZeff of PVP	0.86 (0.84, 0.87)	0.83 (0.80, 0.84)	− 4.149	< 0.001
λHU of PVP	2.48 ± 0.66	1.69 ± 0.44	3.354	< 0.001
ECVf (%*)	34.70 (31.72, 37.87)	26.15 (20.97, 31.02)	− 5.197	< 0.001

The % value reflects the composition ratio

The %* value reflects the percentages

F/Z/χ^2^ values represent the statistics of two independent sample *t*-tests for normal distribution, two independent sample *t*-tests for non-normal distribution and chi-square test, respectively

*AP* arterial phase, *DECT* dual-energy computed tomography, *ECVf* the extracellular volume fraction, *LN* lymph node, *NIC* normalized iodine concentration, *NZeff* normalized effective atomic number, *PVP* portal venous phase, *λHU* slope of the spectral Hounsfield unit curve

### Development of prediction models for Ki-67 expression

Multivariate logistic regression analysis was used to analyse all variables with *p* < 0.05 (Table 3). The CT-reported regional LN status (OR = 5.787; 95% CI: 1.392–24.066; *p* = 0.016), NIC of PVP (OR = 0.170; 95% CI: 0.044–0.656; *p* = 0.010) and ECVf (OR = 0.879; 95% CI: 0.782–0.988; *p* = 0.030) were screened out through forward stepwise logistic regression. The Radiology, DECT and DECT–Radiology models were then established on the basis of the independent risk factors. The Radiology, DECT and DECT–Radiology models were severally proved independently in the validation cohort.

**Table 3:** Forward stepwise logistic regression analysis for the significant variables of radiology and DECT as predicting the expression of Ki-67 of PDAC in the training cohort

Variables	Odds ratio	95% CI	*p *value
CT-reported regional LN status	5.787	1.392–24.066	0.016
NIC of PVP	0.170	0.044–0.656	0.010
ECVf (%)	0.879	0.782–0.988	0.030

*DECT* dual-energy computed tomography, *ECVf* the extracellular volume fraction, *LN* lymph node, *NIC* normalized iodine concentration, *PVP* portal venous phase

### Diagnostic performances of the three models

**Fig. 2:**
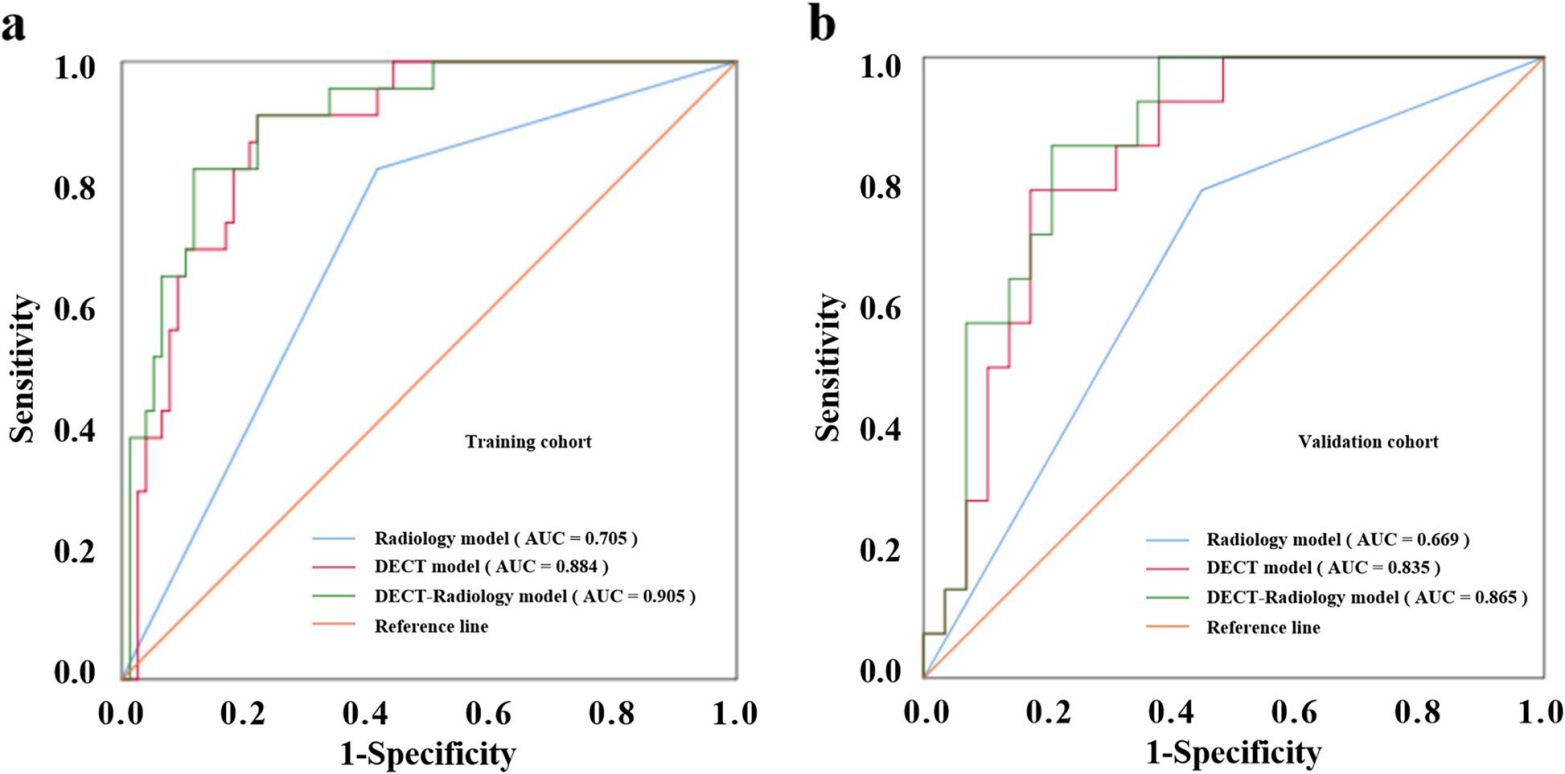
ROCs of Radiology model, DECT model, and DECT–Radiology model for predicting the expression of Ki-67 of PDAC in the training and validation cohorts (**a**, **b**). AUC, area under the curve; ROC, receiver operating characteristic curve; DECT, dual-energy computed tomography

**Fig. 3:**
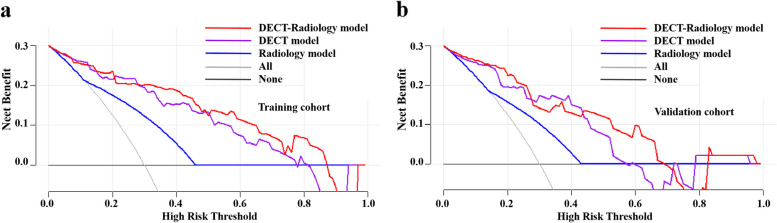
DCA outcomes for the models of Radiology, DECT, and DECT–Radiology. The *y*-axis and *x*-axis demonstrate the net benefit and threshold probability respectively. The black line represents all cases that are assumed to be high Ki-67 expression, and the grey line represents all cases that are assumed to be low Ki-67 expression. The DCA demonstrated that DECT–Radiology model was more advantageous than the Radiology model and DECT model (**a**, **b**). DCA, decision curve analysis; DECT, dual-energy computed tomography

Predictive outcomes of the three models are shown in Table 4. Figures 2 and 3 display the ROC and DCA to identify low and high Ki-67 expression in the two cohorts for the three models. The Radiology model produced moderate diagnostic performances of Ki-67 expression in PDAC patients in the training and validation cohorts, with AUCs of 0.705 (95% CI: 0.590–0.820) and 0.669 (95% CI: 0.499–0.839), respectively. Both the DECT model and the DECT–Radiology model produced preferable diagnostic performances in distinguishing the Ki-67 proportion in PDAC patients, with AUCs of 0.884 (95% CI: 0.816–0.952) and 0.905 (95% CI: 0.826–0.883) in the training cohort. Correspondingly, the DECT model and DECT–Radiology model had AUCs of 0.835 (95% CI: 0.715–0.955) and 0.865 (95% CI: 0.757–0.972), respectively, in the validation cohort. The DeLong test revealed that the DECT–Radiology model performed better than the Radiology model in the training (*p* < 0.001). The diagnostic presentation of the DECT model improved compared with that of the Radiology model (*p* = 0.001) in the training cohort, however, there were nonexistent statistically significant differences in the validation cohort (*p* = 0.093). DCA findings revealed that the DECT–Radiology model could produce a higher net benefit for the ranges of the 0.04–0.90 and 0.04–0.70 threshold probabilities in the training and validation cohorts, respectively.

**Table 4:** Diagnostic performance of the Radiology, DECT and DECT–Radiology models in the training and validation cohorts

Models	Training cohort				Validation cohort			
	AUC (95% CI)	SEN	SPE	DeLong	AUC (95% CI)	SEN	SPE	DeLong
Radiology model	0.705 (0.590–0.820)	0.826	0.587	0.001^a^	0.669 (0.499–0.839)	0.786	0.552	0.093^a^
DECT model	0.884 (0.816–0.952)	0.913	0.779	0.300^b^	0.835 (0.715–0.955)	0.786	0.828	0.514^b^
DECT–Radiology model	0.905 (0.841–0.968)	0.826	0.883	< 0.001^c^	0.865 (0.757–0.972)	0.857	0.793	0.003^c^

*AUC* area under the curve, *CI* confidence interval, *DECT* dual-energy computed tomography, *SEN* sensitivity, *SPE* specificity

^a^Radiology model versus DECT model

^b^DECT model versus DECT–Radiology model

^c^DECT–Radiology versus Radiology model

### Development and performance of DECT–radiology nomogram

**Fig. 4:**
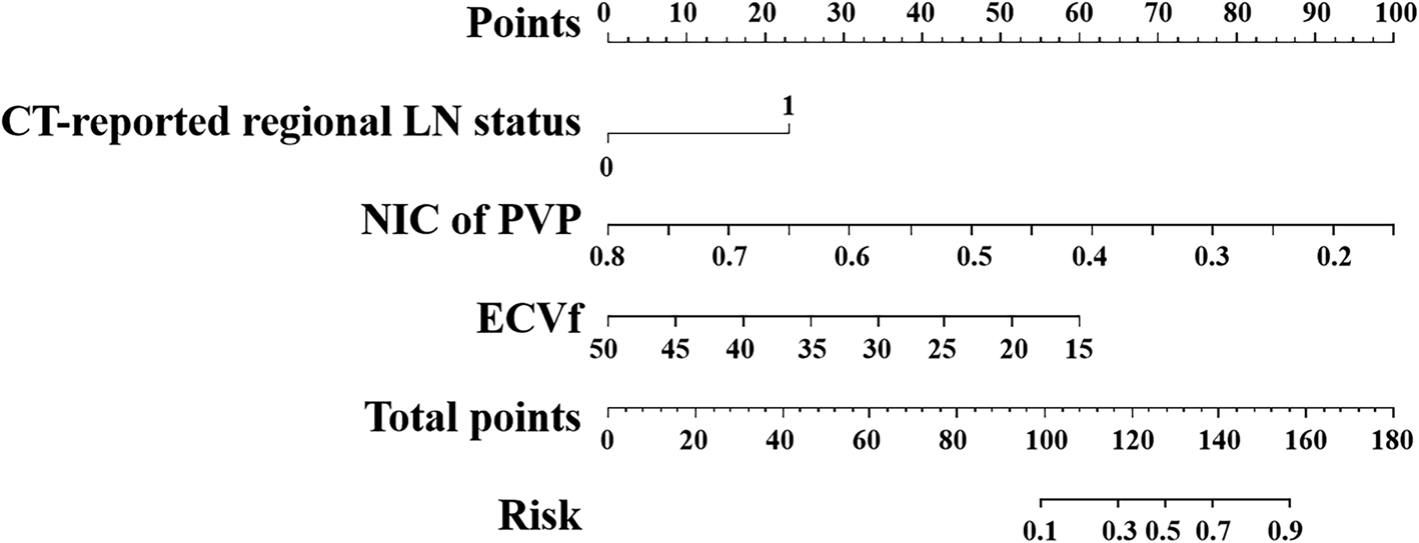
DECT–Radiology nomogram was diagramed uniting one radiological and two DECT variables. The points of each predictive index are scored by the corresponding values on “point line”. The total scores are obtained from the summation of the points of all predictive indexes to assess the Ki-67 expression in PDAC. CT, computed tomography; DECT, dual-energy computed tomography; ECVf, the extracellular volume fraction; LN, lymph node; NIC, normalized iodine concentration; PDAC, pancreatic ductal adenocarcinoma; PVP, portal venous phase

**Fig. 5:**
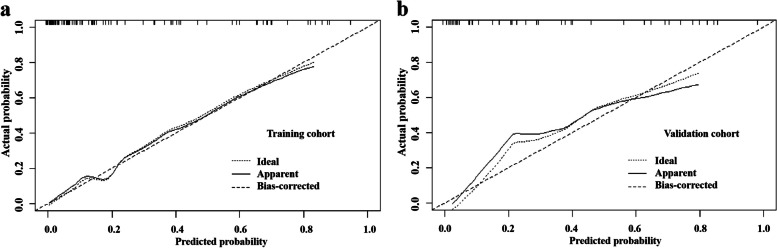
Calibration curves of DECT–Radiology nomogram in cohorts of training and validation (**a**, **b**). Diagonal implies DECT–Radiology nomogram of an ideal performance. Closer to the diagonal implies more accuracy. DECT, dual-energy computed tomography

The DECT–Radiology nomogram was established based on the DECT–Radiology model that possessed the highest diagnostic efficacy for anticipating the expression of Ki-67 in PDAC (Fig. 4). The parameters of the DECT–Radiology nomogram including the CT-reported regional LN status, NIC of PVP and ECVf. The formula was as follows: DECT–Radiology nomogram = 1.787*CT-reported regional LN status–11.921*NIC of PVP–0.133*ECVf + 6.294. A good consistency between the predicted and actual probabilities of the nomogram was observed in the calibration curve analysis in predicting the level of Ki-67 in PDAC in the training and validation cohorts (Fig. 5).

**Fig. 6:**
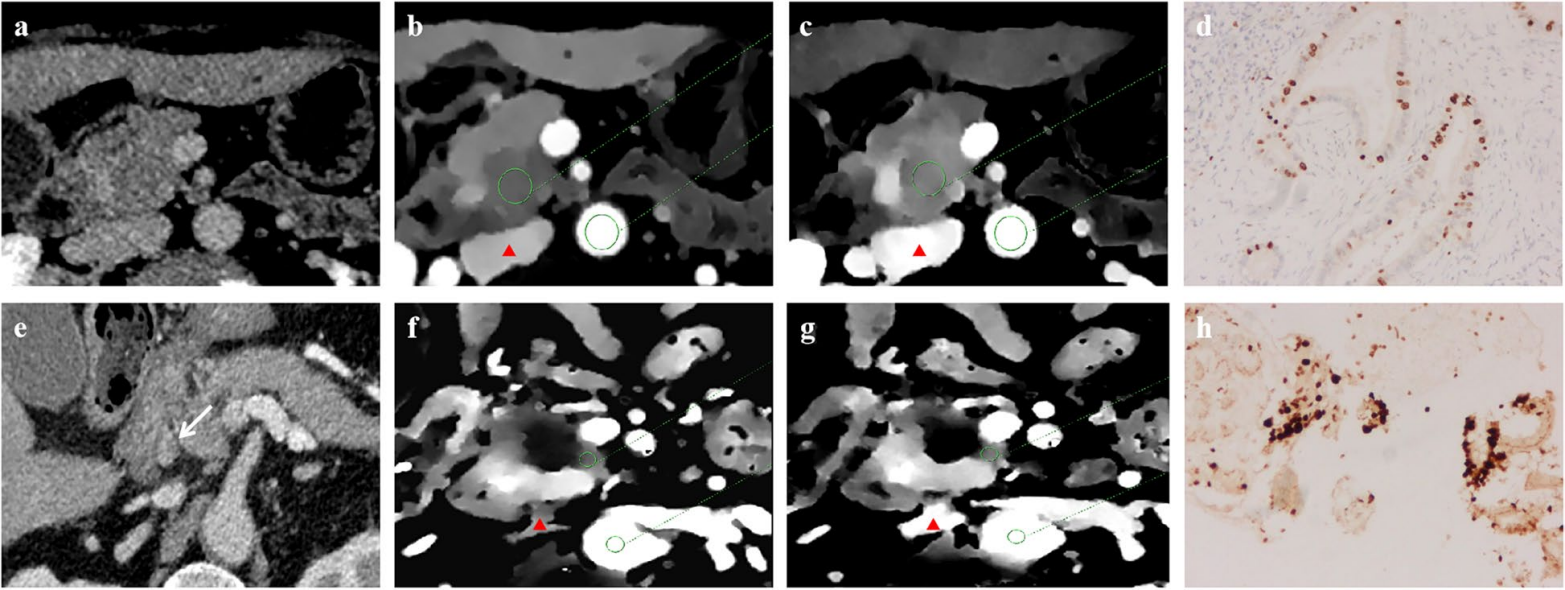
DECT figures of a 66-year-old female patient with low Ki-67 expression in PDAC. **a** CT-reported regional LN negative. **b**, **c** IC_tumour_ and IC_aorta_ in the same layer in the PVP and EP, the NIC of PVP was 0.45 mg/ml and the ECVf was 42.29%. **d** Ki-67 was 30%. (red arrow: inferior vena cava). DECT figures of a 73-year-old male patient with high Ki-67 expression in PDAC. **e** CT-reported regional LN positive. **f**, **g** IC_tumour_ and IC_aorta_ in the same layer in the PVP and EP, the NIC of PVP was 0.34 mg/ml and the ECVf was 37.08%. **h** Ki-67 expression was 60%. (red arrow: inferior vena cava). DECT, dual-energy computed tomography; ECVf, extracellular volume fraction; EP, equilibrium phase; IC, iodine concentration; LN, lymph node; NIC, normalized iodine concentration; PDAC, pancreatic ductal adenocarcinoma; PVP, portal vein phase

Representative images are shown in Fig. 6.

## Discussion

In this retrospective study, a DECT–Radiology model consisting of CT-reported regional LN status, NIC of PVP, and ECVf was developed and validated to achieve the noninvasive prediction of the degree of Ki-67 expression in PDAC. The level of Ki-67 expression was positively correlated with PDAC tumour grade and ultimately disease stage [21]. For high-stage PDAC patients, neoadjuvant therapy (NAC) can prolong disease-specific survival and disease-free survival [22]. Therefore, PDAC patients with high levels of Ki-67 expression may benefit from receiving aggressive NAC. The nomogram derived from the DECT–Radiology model demonstrated better identification ability than the Radiology and DECT models in both the training and validation cohorts in predicting Ki-67 expression, which may be an effective imaging tool to preoperatively and noninvasively identify PDAC patients with high levels of Ki-67 expression.

We discovered that the CT-reported regional LN status was the most important radiological feature to distinguish the degree of Ki-67 expression in PDAC. A higher positive rate of CT-reported regional LN status was found in the high Ki-67 expression set than in the low Ki-67 expression set (82.6% vs 41.5%). The Radiology model was constructed based on this independent indicator and achieved moderate diagnostic effectiveness, with AUCs reaching 0.705 and 0.669 in the training and validation cohorts, respectively. Departed studies have pointed out that PDAC patients with higher levels of Ki-67 expression were more prone to regional LN metastasis [10]. It has been shown that CT-reported LN status significantly correlates with pathological status [23]. The reason that CT-reported regional LN status could predict Ki-67 expression may be because Ki-67 reflects the degree of tumour proliferation activity, and a higher degree of expression indicates that the tumour has more active tumour proliferation and is more likely to infiltrate through the wall of lymphatic vessels and develop lymph node metastasis. However, the judgement of LN metastasis in CT was mainly based on the enhanced and margin status and other indicators, which had certain subjective dependence. Previous studies have discovered the low accuracy of CT for assessing peripancreatic LNM (63%-81%), which was similarly confirmed in our study (66.7%) [24, 25]. Although some radiological features such as parenchyma atrophy and extrapancreatic perineural invasion status have been documented as significant prognostic indicators of PDAC, there were no statistical differences between high and low Ki-67 expression sets and still inability to be used as predictors of Ki-67 expression. This indicated that it was insufficient to assess the Ki-67 expression level by relying on morphological characteristics alone, and blood supply characteristics or internal characteristics may provide comprehensive information.

In this study, our results also indicated that the NIC of PVP and ECVf were independent risk factors for predicting the expression of Ki-67 in PDAC in the series of DECT quantitative parameters. The parameter NIC was obtained through normalized IC, which minimized interindividual hemodynamic variations, and could quantitatively show the extent of tumour neovascularization and objectively reflect iodine deposition in tissues [26]. In our study, the NIC of PVP in the Ki-67 high expression set was significantly lower than that in the Ki-67 low expression set in the training cohort (0.33 vs. 0.24, *p* < 0.001). The possible reasons for this were the fact that high Ki-67 expression represents active cell proliferation, whereas ischaemia and hypoxia due to active cell proliferation in PDAC constitute the microenvironment for the growth of PDAC and that PDAC is a less vascular tumour with a high fibrous stromal component [27]. The ECVf is a quantitative indicator of the extravascular space and extracellular space, and the extravascular space and extracellular space are important parts of the cellular microenvironment. The evolution of tumours is accompanied by changes in the extracellular microenvironment, so the extracellular matrix can partly reflect the development of disease. In our study, the ECVf was significantly lower than that in the low Ki-67 expression set (26.15% vs 34.80%, *p* < 0.001). A previous study revealed that low ECVf indicated poor vascularization for those more prone to hypoxia, which may explain why ECVf could predict the expression status of Ki-67 [15]. The DECT model built on the basis of the NIC of PVP and ECVf had a better diagnostic ability than the Radiology model, in which the AUCs were 0.884 and 0.835 for the training and validation cohorts, respectively.

By incorporating the CT-reported regional LN status, NIC of PVP and ECVf, we established the DECT–Radiology model that emerged with the highest diagnostic efficacy (training cohort: 0.905; validation cohort: 0.865) and net benefits through comparison with the Radiology model and the DECT model. This DECT–Radiology model showed significantly better diagnostic efficacy for the expression of Ki-67 in patients with PDAC than the Radiology model in the training and validation cohorts (all *p* < 0.05, DeLong test). The DECT–Radiology nomogram, a simple, potentially reliable and reproducible tool, was established on the basis of the DECT–Radiology model and possessed the highest diagnostic ability for predicting Ki-67 expression in PDAC. Good agreement between prediction and observation was demonstrated in the calibration curve in both the training cohort and validation cohort.

This study had some limitations. First, this study was retrospective, and the selection of samples may still exist in a biased manner despite strict inclusion and exclusion criteria. Second, because the sample scale of this study was small and conducted in one infirmary, further multicentre large-scale studies are needed to verify the findings before the DECT–Radiology nomogram can be applied in clinical practice. Finally, some of the samples in this study were puncture biopsies, which were not fully representative of all tumour tissues due to tumour heterogeneity and may lead to some bias in Ki-67 measurements. Most PDAC patients were unable to undergo radical resection to obtain a surgical specimen at the advanced stage at the time of initial medical consultation, whereas puncture biopsy could assist in individualized clinical decision-making.

## Conclusion

In conclusion, a model that combines key radiological features and dual-energy computed tomography quantitative parameters is helpful in the preoperative and noninvasive evaluation of the Ki-67 expression in patients with PDAC. The visualized nomogram constructed based on the DECT–Radiology model is simple and effective and may help clinicians to screen out PDAC patients with high Ki-67 expression.

### Supplementary Information

**Additional file 1.**

## Data Availability

The data underlying this article cannot be shared publicly for the privacy of individuals who participated in the study. The data will be provided by the corresponding author on sensible request.

## Abbreviations

AP: Arterial phase
AUC: Area under the curve
CI: Confidence interval
DCA: Decision curve analysis
DECT: Dual-energy computed tomography
ECVf: Extracellular volume fraction
EP: Equilibrium phase
IHC: Immunohistochemistry
LN: Lymph node
NAC: Neoadjuvant therapy
NIC: Normalized iodine concentration
NZeff: Normalized effective atomic number
PDAC: Pancreatic ductal adenocarcinoma
PVP: Portal venous phase
ROC: Receiver operating characteristic curve
SD: Standard deviation
λHU: Slope of the spectral attenuation curves
