# Supplementary material for: Development and validation of a model for predicting the expression of Ki-67 in pancreatic ductal adenocarcinoma with radiological features and dual-energy computed tomography quantitative parameters
Source: Insights Imaging. 2024 Feb 14;15:41. doi: 10.1186/s13244-024-01617-8 (PMC10866831; doi:10.1186/s13244-024-01617-8)
Supplement: Supplementary file 1 — Additional file 1. [file 13244_2024_1617_MOESM1_ESM.docx]

**Development and validation of a model for predicting the expression of Ki-67 in pancreatic ductal adenocarcinoma with radiological features and dual-energy computed tomography quantitative parameters**

**ELECTRONIC SUPPLEMENTARY MATERIAL**

**Abbreviations:**

AJCC American Joint Committee on Cancer

AP Arterial phase

CA Celiac axis

CT Computed tomography

EP Equilibrium phase

HA Arteriae hepatica propria

PDAC Pancreatic ductal adenocarcinoma

PVP Portal vein phase

ROI Regions of interest

SMA Superior mesenteric artery

**Materials and methods**

**DECT image acquisition**

Contrast–enhanced abdominal scans were performed using a DECT (IQon spectral CT, Philips Healthcare) with craniocaudal supine position. The equal scan protocol was as follows: tube voltage, 120 kV; smart mAs; turn-around time, 0.5 s; detector layers, 64 × 0.625 mm; matrix, 512 × 512; reconstruction thicknesses, 1.25 mm. Patients were intravenously injected with non-ionic contrast media (Omnipaque, 350 mgI/ml) in a dosage of 1.5 ml/kg at 3.5 ml/s using high–pressure automatic injector, then pumped 30 ml saline at the equal pace. Arterial phase (AP), portal vein phase (PVP) and equilibrium phase (EP) imaging were executed 10 s, 40 s, and 180 s after the predefined critical value of 150 HU in aorta abdominalis (activated bolus tracking), respectively.

**Radiology and DECT candidate variables**

The radiological features of PDAC included tumour location, CT-reported T stage, CT-reported regional LN status, atrophy of the pancreatic parenchyma, expansion of the main pancreatic duct, blood vessels invasion and extrapancreatic perineural invasion. The left wall of the superior mesenteric vein was taken as the boundary, the right as the head and neck and the left as the body and tail. In light of the American Joint Committee on Cancer (AJCC) standard, a maximum diameter of tumour ≤ 4cm was defined as T1–T2 and a maximum diameter > 4cm or the celiac axis (CA), superior mesenteric artery (SMA) or arteriae hepatica propria (HA) was defined as T3–T4[1]. Regional LNs of PDAC with short axial diameter > 10mm, uneven density, blurred margins, insignificant enhancement, internal necrosis or mutual fusion were considered as the CT-reported regional LN status positive; otherwise, CT-reported regional LN negative[2, 3]. Pancreatic parenchymal atrophy was defined as an anteroposterior diameter of the pancreas after subtracting the pancreatic duct < 15mm[4]. A main pancreatic duct diameter > 2.5mm was considered to indicate dilation[5]. Vascular invasion was defined as stenosis or occlusion of the vascular lumen or tumour–vascular contact surface ≥ 180°[6]. The CA, SMA, HA, splenic artery, superior mesenteric vein and portal vein of PDAC patients were evaluated[7]. The status of extrapancreatic perineural invasion was defined as positive when extrapancreatic soft-tissue infiltration was identified extending directly from the intrapancreatic tumour along any of the four established pathways for perineural spread of PDAC[8]. In cases of disagreement, the two imaging diagnostic physicians reached an agreement through consultation and discussion.

The DECT quantitative parameters of PDAC included NIC, NZeff and λHU in the AP and PVP. Owing to the spectral curve showing greater instability when its energy was over 100 keV, 40–100 keV was chosen to calculate λHU, which was calculated as follows: λHU = (HU40keV–HU100keV)/(100–40) [9]. The extracellular volume fraction (ECVf) was measured in the EP, which was calculated as follows: ECVf = (1−hematocrit)×(ΔHUtumour/ΔHUaorta) × 100, where ΔHUtumour and ΔHUaorta were Hounsfield units in the equilibrium phase minus the Hounsfield units before administering the contrast agent to the tumour and aorta, respectively. The hematocrit were obtained from the patients' clinical medical records[10]. The area of interest (ROI) was placed within the PDAC lesion that may contain the largest lesion area to avoid significant necrosis, cysts and vascular areas. Across the different phases, ROI size, shape, and position remained consistent via the copy-and-paste function. Qualitative and quantitative analyses were executed by two radiologists who finished homogenization training in abdominal imaging, had sustained training for 8 and 17 years and were unaware of the pathological findings.

**References：**

[1]Chun YS, Pawlik TM, Vauthey JN. 8th Edition of the AJCC Cancer Staging Manual: Pancreas and Hepatobiliary Cancers. Ann Surg Oncol. 2018 Apr;25(4):845-847. [https://doi.org/10.1245/s10434–017–6025–x.](https://doi.org/10.1245/s10434-017-6025-x%5d)

[2]Dorfman RE, Alpern MB, Gross BH, Sandler MA. Upper abdominal lymph nodes: criteria for normal size determined with CT. Radiology. 1991 Aug;180(2):319-22. <https://doi.org/10.1148/radiology.180.2.2068292>.

[3]Efremidis SC, Vougiouklis N, Zafiriadou E, et al. Pathways of lymph node involvement in upper abdominal malignancies: evaluation with high-resolution CT. Eur Radiol. 1999;9(5):868-74. https://doi.org/10.1007/s003300050757.

[4]Shi C, Siegelman SS, Kawamoto S, et al. Pancreatic duct stenosis secondary to small endocrine neoplasms: a manifestation of serotonin production? Radiology. 2010 Oct;257(1):107-14. <https://doi.org/10.1148/radiol.10100046.>

[5]Tanaka S, Nakao M, Ioka T, et al. Slight dilatation of the main pancreatic duct and presence of pancreatic cysts as predictive signs of pancreatic cancer: a prospective study. Radiology. 2010 Mar;254(3):965-72.  https://doi.org/10.1148/radiol.09090992.

[6]Al-Hawary MM, Francis IR, Chari ST, et al. Pancreatic ductal adenocarcinoma radiology reporting template: consensus statement of the society of abdominal radiology and the american pancreatic association. Gastroenterology. 2014 Jan;146(1):291-304.e1. <https://doi.org/10.1053/j.gastro.2013.11.004>.

[7]Buchs NC, Chilcott M, Poletti PA, Buhler LH, Morel P. Vascular invasion in pancreatic cancer: Imaging modalities, preoperative diagnosis and surgical management. World J Gastroenterol. 2010 Feb 21;16(7):818-31. <https://doi.org/10.3748/wjg.v16.i7.818>.

[8]Chang ST, Jeffrey RB, Patel BN, et al. Preoperative Multidetector CT Diagnosis of Extrapancreatic Perineural or Duodenal Invasion Is Associated with Reduced Postoperative Survival after Pancreaticoduodenectomy for Pancreatic Adenocarcinoma: Preliminary Experience and Implications for Patient Care. Radiology. 2016 Dec;281(3):816-825. https://doi.org/10.1148/radiol.2016152790.

[9]Luo YH, Mei XL, Liu QR, et al. Diagnosing cervical lymph node metastasis in oral squamous cell carcinoma based on third-generation dual-source, dual-energy computed tomography. Eur Radiol. 2023 Jan;33(1):162-171. [https://doi.org/10.1007/s00330–022–09033–6.](https://doi.org/10.1007/s00330-022-09033-6.)

[10]Fukukura Y, Kumagae Y, Higashi R, et al. Extracellular volume fraction determined by equilibrium contrast-enhanced dual-energy CT as a prognostic factor in patients with stage IV pancreatic ductal adenocarcinoma. Eur Radiol. 2020 Mar;30(3):1679-1689. [https://doi.org/10.1007/s00330–019–06517–w](https://doi.org/10.1007/s00330-019-06517-w).
